# Supplementary material for: Capturing a rhodopsin receptor signalling cascade across a native membrane
Source: Nature. 2022 Apr 6;604(7905):384–90. doi: 10.1038/s41586-022-04547-x (PMC9007743; doi:10.1038/s41586-022-04547-x)
Supplement: Supplementary file 1 — This file contains Supplementary Figure 1: Uncropped gel image of the soluble fraction; Supplementary Notes for Extended Data Figure 3; and Supplementary Table 1: Proteomics of purified bovine ROS disc membrane. [file 41586_2022_4547_MOESM1_ESM.pdf]

nature portfolio

https://doi.org/10.1038/s41586-022-04547-x

Supplementary information

Capturing a rhodopsin receptor signalling cascade across a native membrane

In the format provided by the authors and unedited

Supplementary Information

Capturing a rhodopsin receptor signalling cascade across a native membrane

Siyun Chen[1,2], Tamar Getter[3], David Salom[3], Di Wu[1,2], Daniel Quetschlich[1,2], Dror S. Chorev[1*], Krzysztof Palczewski[3,4*], Carol V. Robinson[1,2*].

1. Chemical Research Laboratory, University of Oxford, South Path Parks Road Oxford, UK.

2. Kavli Institute for Nanoscience Discovery, University of Oxford, South Parks Road, Oxford, OX1 3QU, UK.

3. Gavin Herbert Eye Institute, Department of Ophthalmology, University of California, Irvine, Irvine, CA, USA

4. Departments of Physiology and Biophysics, Chemistry, Molecular Biology and Biochemistry, University of California, Irvine, Irvine, CA, USA.

Contents:

p2: Supplementary Figure 1 Uncropped gel image of the soluble fraction.

p3: Supplementary video Legend of the experimental set-up.

p4: Supplementary notes for Extended Data Figure 3

p6: Supplementary table 1 Proteomics of purified bovine ROS disc membrane.

Supplementary Figure 1

Unedited gel from Extended Data Fig. 7a. Separation of the component subunits of the Gt and PDE6 complex on a 2D page gel, pre-stained with protein standards.

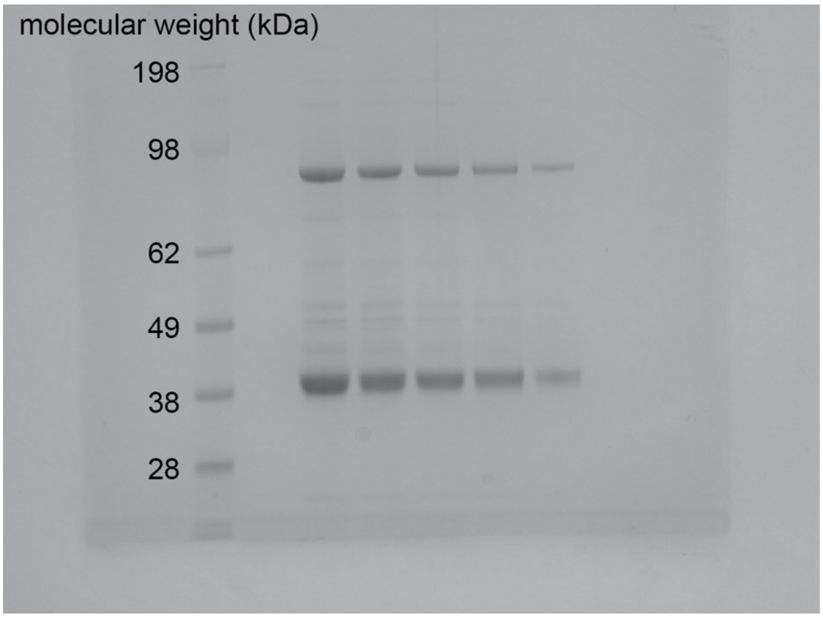

Supplementary Video

A video of the experimental set-up begins with the positioning of the gold-coated nanoflow capillary close to the source aperture of the UHMR mass spectrometer. Red-light conditions are employed at this time while we establish a stable nanoflow electrospray mass spectrum for rho/opsin from native membrane vesicles. Once a stable signal is established, the capillary is illuminated with white light and the photoconversion is initiated. The rho/opsin ratio can be seen to transition from rho to the lower molecular mass species (opsin) as a function of real-time light exposure. Expansion across charge states in the mass spectrum shows the two peaks assigned to rho/opsin, with the same charge state, revealing the changing population as a function of time. In the mass spectral recording shown here, a pulse of light is applied for 40 s approximately 5 min after initial exposure to light. At this time point opsin is predominant and increases relative to rho during the 40 s duration of the light pulse.

Supplementary Notes for Extended Data Figure 3

A natural logarithm plot of rho abundance against time fits to a straight line in the period of 18 s to 300 s (R^2=0.94 ,0.98, 0.98 from 3 replicate experiments) in disc membrane vesicles and 18 s to 180 s (R^2=0.95, 0.96, 0.99) for the faster reaction in LMNG micelles. Because at the beginning of hydrolysis, the concentration of all- *trans*-retinal remains low, and the contribution from regeneration > 18 s is negligible in the system studied here, hydrolysis is the rate-limiting, and dominant reaction, such that it can be considered as irreversible. k_1 and k_2 are referred to as k_{iso} and k'_{hyd} in this phase respectively.

Experimentally, we first independently obtained the rate constant k_{iso}. This rate constant is derived from the experiment in which *cis*-retinal rho isomerisation is measured after addition of hydroxylamine and is represented in our kinetic model as the isomerization of *cis*-retinal rho to rho* at a rate k_{iso} including a minimal contribution of regeneration.

Since this is a first-order reaction, the rate can be expressed as follows:
d[*cis*-retinal rho]/dt = - k_{iso}[*cis*-retinal rho] (eq1)
where [*cis*-retinal rho] is the concentration of *cis*-retinal rho and t is the illumination time.

The solution can be written as follows: [*cis*-retinal rho]=[*cis*-retinal rho]1 exp(-k_{iso} (t-t1) (eq2)

where t1 is the initial time point of this data subset and [*cis*-retinal rho]1 is the concentration of *cis*-retinal rho at t=t1.

Equation 2 can be used to fit the experimental data of the rho photoconversion in the presence of hydroxylamine. In this case rho only consists of *cis*-retinal rho, and excludes rho*. Plotting the abundance of *cis*-retinal rho as a function of time gives the fitting parameter in Equation 2: k_{iso}.

Next, under normal photoconversion conditions, the intermediate rho* is isomerized from *cis*-retinal rho at a rate k_{iso} and slowly hydrolyses (k'_{hyd}) into opsin and all-*trans*-retinal at a rate expressed as follows:

d[rho*]/dt = k_{iso}[*cis*-retinal rho] – k'_{hyd}[rho*] (eq3)

where [rho*] is the abundance of all-*trans*-retinylidene-opsin, t is the illumination time.

substituting equation 2 into 3, yields a differential equation with the solution:

[rho*] = $k_{iso}/(k'_{hyd}-k_{iso})$ {exp(-k_{iso}(t-t1)) - exp(-k'_{hyd}(t-t1))} [*cis*-retinal rho]1 + [rho*]1 (eq4)

Where t1 is the initial time point of this data subset and [rho*]1 is the abundance of all-*trans*-retinal rho at t=t1. [rho*] is obtained from the difference between the measured abundance of rho under normal conditions and the abundance of *cis*-retinal rho from Equation 2.

The abundance of rho* as a function of time. k_{iso} can be determined independently using Equation 2. The fitting parameters in Equation 4 gives k'_{hyd}.

Finally, in this kinetic model, [rho] can be expressed as:
[rho]=$k'_{hyd}/(k'_{hyd} - k_{iso})$ exp(-k_{iso} t) - $k_{iso}/(k'_{hyd} - k_{iso})$ exp(-k'_{hyd} t)}[*cis*-retinal rho]0 (eq5)

Since at all times [cis-retinal rho]+[rho*]+[ops]=[total], [ops] can be expressed as:

[ops]= {k_{iso} exp(-k'_{hyd} t) – k'_{hyd} exp(-k_{iso} t)}/(k'_{hyd} –k_{iso}) [*cis*-retinal rho]0+[ops]0 (eq6)

Since $k_{iso} >> k'_{hyd}$, Equation 5 and Equation 6 can be simplified as follows:
[rho]=[*cis*-retinal rho]0 exp(-k_{hyd} t) (eq7)
[ops]={1-exp(-k_{hyd} t)}[*cis*-retinal rho]0+[tot]-[*cis*-retinal rho]0 (eq8)

t is illumination time. [rho] is the measured abundance of rho under normal experimental conditions. [ops] is the measured abundance of opsin under normal conditions. [*cis*-retinal rho]0 is the abundance of *cis*-retinal rho from the dark-adapted membrane and regenerated from opsin before this phase. Total concentration of *cis*-retinal rho, rho* and ops, [tot] =100% (percentage abundance is used throughout). Since [*cis*-retinal rho]0 is difficult to estimate or measure we obtain this from [rho] as a function of time and the fitting parameter k_{hyd} yields [*cis*-retinal rho]0.

Thus, under normal conditions, k_{hyd} can be determined first from this phase using Equation 7 or Equation 8. With the additional data set, in the presence of hydroxylamine, k_{iso} can be determined independently using Equation 2. Furthermore, the rate constant of hydrolysis literally from rho*, k'_{hyd} is refined using Equation 4.

Isomerization of cis-retinal rho to rho* goes to completion as hydrolysis takes place and the concentration of all-*trans*-retinal increases. In our kinetic model (**d** above), hydrolysis of the rho* Schiff base is a reversible second order reaction. Additionally, from ~420 s onwards, the concentration of opsin was not observed to reach equilibrium but rather continues to rise slowly. A plausible explanation is that as hydrolysis proceeds slowly, all-*trans*-retinal is slowly converted, *via* regenerative isomerization, to *cis*-retinal and combines with opsin.

Accession	Mass	Num. of matches	Num. of significant matches	Num. of sequences	Num. of significant sequences	Description
tr|F1MWM0|F1MWM0_BOVIN	259606	23	15	13	8	ATP binding cassette subfamily A member 4 OS=Bos taurus OX=9913 GN=ABCA4 PE=4 SV=2
sp|P00829|ATPB_BOVIN	56249	20	17	15	12	ATP synthase subunit beta, mitochondrial OS=Bos taurus OX=9913 GN=ATP5F1B PE=1 SV=2
sp|P02699|OPSD_BOVIN	39552	15	10	6	5	Rhodopsin OS=Bos taurus OX=9913 GN=RHO PE=1 SV=1
tr|F1MLW4|F1MLW4_BOVIN	140140	15	13	11	9	Retinol-binding protein 3 OS=Bos taurus OX=9913 GN=RBP3 PE=4 SV=2
tr|E1BJB1|E1BJB1_BOVIN	50274	7	5	4	3	Tubulin beta chain OS=Bos taurus OX=9913 GN=TUBB2A PE=1 SV=1
tr|F1MZV1|F1MZV1_BOVIN	103425	8	7	6	5	Hexokinase-1 OS=Bos taurus OX=9913 GN=HK1 PE=1 SV=2
sp|Q2HJ86|TBA1D_BOVIN	50935	8	7	5	4	Tubulin alpha-1D chain OS=Bos taurus OX=9913 GN=TUBA1D PE=1 SV=1
sp|P52205|ROM1_BOVIN	37965	12	10	9	7	Rod outer segment membrane protein 1 OS=Bos taurus OX=9913 GN=ROM1 PE=1 SV=2
sp|Q9BGI7|OPSR_BOVIN	41185	5	4	4	3	Long-wave-sensitive opsin 1 OS=Bos taurus OX=9913 GN=OPN1LW PE=2 SV=1
sp|Q28139-2|NCKX1_BOVIN	130174	8	6	4	3	Isoform 2 of Sodium/potassium/calcium exchanger 1 OS=Bos taurus OX=9913 GN=SLC24A1
sp|P0C0S9|H2A1_BOVIN	14083	5	5	1	1	Histone H2A type 1 OS=Bos taurus OX=9913 PE=1 SV=2
sp|Q3T100|MGST3_BOVIN	17101	5	3	2	2	Microsomal glutathione S-transferase 3 OS=Bos taurus OX=9913 GN=MGST3 PE=2 SV=1
sp|P08168|ARRS_BOVIN	45418	11	9	9	7	S-arrestin OS=Bos taurus OX=9913 GN=SAG PE=1 SV=1
tr|F1MLB8|F1MLB8_BOVIN	59767	7	5	6	4	ATP synthase subunit alpha OS=Bos taurus OX=9913 GN=ATP5F1A PE=1 SV=1
sp|P80724|BASP1_BOVIN	22997	4	4	3	3	Brain acid soluble protein 1 OS=Bos taurus OX=9913 GN=BASP1 PE=1 SV=3
sp|Q2HJ97|PHB2_BOVIN	33337	3	2	2	1	Prohibitin-2 OS=Bos taurus OX=9913 GN=PHB2 PE=2 SV=1
tr|F1MRD0|F1MRD0_BOVIN	42167	3	2	2	1	Actin, cytoplasmic 1 OS=Bos taurus OX=9913 GN=ACTB PE=3 SV=2
tr|E1BME9|E1BME9_BOVIN	51664	2	2	1	1	NADH dehydrogenase [ubiquinone] flavoprotein 3, mitochondrial OS=Bos taurus OX=9913 GN=NDUFV3 PE=1 SV=2
tr|F1N0X5|F1N0X5_BOVIN	39839	6	5	6	5	Peripherin-2 OS=Bos taurus OX=9913 GN=PRPH2 PE=4 SV=1
sp|P04695|GNAT1_BOVIN	40396	5	4	4	3	Guanine nucleotide-binding protein G(t) subunit alpha-1 OS=Bos taurus OX=9913 GN=GNAT1 PE=1 SV=3
sp|Q00194|CNGA1_BOVIN	79951	5	2	3	1	cGMP-gated cation channel alpha-1 OS=Bos taurus OX=9913 GN=CNGA1 PE=1 SV=1
sp|A2VDL6|AT1A2_BOVIN	113419	5	3	4	2	Sodium/potassium-transporting ATPase subunit alpha-2 OS=Bos taurus OX=9913 GN=ATP1A2 PE=1 SV=1
tr|F1MS25|F1MS25_BOVIN	27999	2	2	1	1	NADH dehydrogenase [ubiquinone] iron-sulfur protein 3, mitochondrial OS=Bos taurus OX=9913 GN=NDUFS3 PE=3 SV=2
tr|A6H783|A6H783_BOVIN	31193	2	2	1	1	VDAC5P protein OS=Bos taurus OX=9913 GN=VDAC5P PE=2 SV=1
sp|P21457|RECO_BOVIN	23376	4	3	2	2	Recoverin OS=Bos taurus OX=9913 GN=RCVRN PE=1 SV=3
tr|F1MBC7|F1MBC7_BOVIN	11975	2	2	1	1	Cytochrome c oxidase subunit 6A, mitochondrial OS=Bos taurus OX=9913 PE=3 SV=1
sp|Q3ZBU2|CISD1_BOVIN	12146	2	2	1	1	CDGSH iron-sulfur domain-containing protein 1 OS=Bos taurus OX=9913 GN=CISD1 PE=1 SV=1
sp|P13620|ATP5H_BOVIN	18738	8	4	4	2	ATP synthase subunit d, mitochondrial OS=Bos taurus OX=9913 GN=ATP5PD PE=1 SV=2
sp|Q08DK4|GHC1_BOVIN	34965	3	1	1	1	Mitochondrial glutamate carrier 1 OS=Bos taurus OX=9913 GN=SLC25A22 PE=2 SV=1
sp|P13619|AT5F1_BOVIN	28860	4	4	3	3	ATP synthase F(0) complex subunit B1, mitochondrial OS=Bos taurus OX=9913 GN=ATP5PB PE=1 SV=2
tr|F1N2J9|F1N2J9_BOVIN	23842	4	2	4	2	Ras-related protein Rab-8B OS=Bos taurus OX=9913 GN=RAB8B PE=4 SV=1
sp|P02698|GBG1_BOVIN	8709	2	2	1	1	Guanine nucleotide-binding protein G(T) subunit gamma-T1 OS=Bos taurus OX=9913 GN=GNGT1 PE=1 SV=2
sp|Q3T165|PHB_BOVIN	29843	3	2	2	2	Prohibitin OS=Bos taurus OX=9913 GN=PHB PE=2 SV=1
sp|P27674|GTR1_BOVIN	54439	2	2	2	2	Solute carrier family 2, facilitated glucose transporter member 1 OS=Bos taurus OX=9913 GN=SLC2A1 PE=1 SV=1
sp|P34943|NDUA9_BOVIN	42879	2	1	2	1	NADH dehydrogenase [ubiquinone] 1 alpha subcomplex subunit 9, mitochondrial OS=Bos taurus OX=9913 GN=NDUFA9 PE=1 SV=1
sp|P32007|ADT3_BOVIN	33084	9	1	6	1	ADP/ATP translocase 3 OS=Bos taurus OX=9913 GN=SLC25A6 PE=1 SV=3
sp|P00423|COX41_BOVIN	19616	4	3	2	2	Cytochrome c oxidase subunit 4 isoform 1, mitochondrial OS=Bos taurus OX=9913 GN=COX4I1 PE=1 SV=1
sp|P55203|GUC2D_BOVIN	121433	6	3	4	2	Retinal guanylyl cyclase 1 OS=Bos taurus OX=9913 GN=GUCY2D PE=1 SV=1
sp|P00426|COX5A_BOVIN	16896	5	4	3	2	Cytochrome c oxidase subunit 5A, mitochondrial OS=Bos taurus OX=9913 GN=COX5A PE=1 SV=2
tr|F1MXX0|F1MXX0_BOVIN	83392	4	2	3	1	MICOS complex subunit MIC60 OS=Bos taurus OX=9913 GN=IMMT PE=1 SV=1
sp|P02721|ATP5J_BOVIN	12524	4	3	2	2	ATP synthase-coupling factor 6, mitochondrial OS=Bos taurus OX=9913 GN=ATP5PF PE=1 SV=2
sp|Q8MJG0|R9BP_BOVIN	25978	1	1	1	1	Regulator of G-protein signaling 9-binding protein OS=Bos taurus OX=9913 GN=RGS9BP PE=1 SV=2
tr|A0A0N4STN0|A0A0N4STN0_BOVIN	11409	2	2	1	1	Uncharacterized protein OS=Bos taurus OX=9913 GN=ATP5MG PE=4 SV=1
sp|P04394|NDUV2_BOVIN	27575	1	1	1	1	NADH dehydrogenase [ubiquinone] flavoprotein 2, mitochondrial OS=Bos taurus OX=9913 GN=NDUFV2 PE=1 SV=3
tr|E1BJA2|E1BJA2_BOVIN	67115	3	1	3	1	Apoptosis inducing factor mitochondria associated 1 OS=Bos taurus OX=9913 GN=AIFM1 PE=1 SV=1
tr|A7Z066|A7Z066_BOVIN	68189	3	2	2	1	Calnexin OS=Bos taurus OX=9913 GN=CANX PE=1 SV=1

Supplementary Table 1

Proteomics of purified bovine ROS disc membrane. For experimental details see methods. This proteomics experiment was performed once.
